# The potential protective effects of pre-injury exercise on neuroimmune responses following experimentally-induced traumatic neuropathy: a systematic review with meta-analysis

**DOI:** 10.3389/fimmu.2023.1215566

**Published:** 2023-09-11

**Authors:** Meghan A. Koop, Marije L. S. Sleijser-Koehorst, Carlijn R. Hooijmans, Paul Q. Tdlohreg, Ivo J. Lutke Schipholt, Gwendolyne G. M. Scholten-Peeters, Michel W. Coppieters

**Affiliations:** ^1^ Faculty of Behavioural and Movement Sciences, Vrije Universiteit Amsterdam, Amsterdam Movement Sciences, Amsterdam, Netherlands; ^2^ Meta Research Team, Department of Anaesthesiology, Pain and Palliative Care, Radboud University Medical Center, Nijmegen, Netherlands; ^3^ Department of Clinical Chemistry, Laboratory Medical Immunology, Amsterdam University Medical Centre, Location VUmc, Amsterdam, Netherlands; ^4^ Menzies Health Institute Queensland, Griffith University, Brisbane, QLD, Australia

**Keywords:** running, prevention, peripheral nerve injury, inflammation, animal model

## Abstract

Pre-clinical evidence shows that neuropathy is associated with complex neuroimmune responses, which in turn are associated with increased intensity and persistence of neuropathic pain. Routine exercise has the potential to mitigate complications of future nerve damage and persistence of pain through neuroimmune regulation. This systematic review aimed to explore the effect of pre-injury exercise on neuroimmune responses, and other physiological and behavioural reactions following peripheral neuropathy in animals. Three electronic databases were searched from inception to July 2022. All controlled animal studies assessing the influence of an active exercise program prior to experimentally-induced traumatic peripheral neuropathy compared to a non-exercise control group on neuroimmune, physiological and behavioural outcomes were selected. The search identified 17,431 records. After screening, 11 articles were included. Meta-analyses showed that pre-injury exercise significantly reduced levels of IL-1β (SMD: -1.06, 95% CI: -1.99 to -0.13, n=40), but not iNOS (SMD: -0.71 95% CI: -1.66 to 0.25, n=82). From 72 comparisons of different neuroimmune outcomes at different anatomical locations, vote counting revealed reductions in 23 pro-inflammatory and increases in 6 anti-inflammatory neuroimmune outcomes. For physiological outcomes, meta-analyses revealed that pre-injury exercise improved one out of six nerve morphometric related outcomes (G-ratio; SMD: 1.95, 95%CI: 0.77 to 3.12, n=20) and one out of two muscle morphometric outcomes (muscle fibre cross-sectional area; SMD: 0.91, 95%CI: 0.27 to 1.54, n=48). For behavioural outcomes, mechanical allodynia was significantly less in the pre-injury exercise group (SMD -1.24, 95%CI: -1.87 to -0.61) whereas no overall effect was seen for sciatic function index. *Post hoc* subgroup analysis suggests that timing of outcome measurement may influence the effect of pre-injury exercise on mechanical allodynia. Risk of bias was unclear in most studies, as the design and conduct of the included experiments were poorly reported. Preventative exercise may have potential neuroprotective and immunoregulatory effects limiting the sequalae of nerve injury, but more research in this field is urgently needed.

## 1 Introduction

Regular exercise is associated with less chronic pain (1) and reduces the risk of several chronic conditions, such as cardiovascular disease, diabetes, metabolic syndrome, and even neurodegenerative diseases (2–5). However, for peripheral neuropathic pain conditions, the preventative effects of exercise are less clear. Exercise is recommended for patients with neuropathic pain (6), but research on the effect of exercise on neuropathic pain has mostly focused on exercise prescribed as a treatment after sustaining the injury (7, 8). A recent review and meta-analysis about the effect of exercise on experimentally-induced neuropathic pain in animals included exercise programs that started both before and after peripheral neuropathy induction (9). Interestingly, this review found that the beneficial effect of exercise on mechanical allodynia was related to the timing of the exercise program, alluding to a benefit of exercise prior to sustaining a neuropathy. Unfortunately, underlying working mechanisms of exercise were not synthesised along with the mechanical allodynia results (9).

Animal experimental studies show that nerve injuries result in a cascade of complex neuroimmune responses involving multiple mediators, such as neuropeptides, cytokines, gene expression and hormones (10–13). However, the balance of pro- and anti-inflammatory responses are integral for the recovery/healing process (14, 15). For example, initial pro-inflammatory responses to nerve damage, such as transient neutrophil activation, are normal, and might even be protective against the development of chronic pain (15, 16). On the other hand, pro-inflammatory responses (such as dorsal root ganglion (DRG) macrophage activation (17), dorsal horn microglia activation (18), and pronociceptive cytokines and chemokines (19)) are responsible for predisposing the development of neuropathic pain (hyperalgesia/allodynia) and persistence of the pain. Furthermore, pain resolution after peripheral nerve injury requires regulatory T cells and M2 macrophages [18]. Altogether, a maladaptive inflammatory response to peripheral nerve injury contributes to the generation of persistence of pain (16). Thus, it seems ideal to prevent an excessive inflammatory response and/or promote the anti-inflammatory resolution phase.

Sedentary behaviour, in addition to injury, promotes and prolongs this pro-inflammatory response via alterations in macrophage phenotype (M1) at the site of injury (20). In addition to immune alterations, sedentary behaviour changes the reactivity of the central nervous system promoting greater excitability and less inhibition (21). Exercise, on the other hand, promotes anti-inflammatory responses (22, 23) increased central nociceptive inhibition and decreased central excitability aiding in healing and analgesia (21, 24). The immunoregulatory effects of exercise may be mediated by multiple mechanisms. Muscular contraction induces cytosolic changes (increased calcium ions, activation of p38 MAPK etc.) which leads to activation of transcription factors (e.g., activation protein (AP)-1) leading to the production of myokines (e.g., IL-6). Together with increased sympathoadrenal activity these responses induce an anti-inflammatory environment with each bout of exercise (22, 25, 26). Routine exercise has the potential to mitigate pain through neuroimmune regulation, as pre-injury exercise in animals prevented hyperalgesia resulting from repeated acidic saline injections by promoting anti-inflammatory mechanisms (27, 28). This suggests that exercise primes the neuroimmune system to an anti-inflammatory state and in turn can reduce the complications of future nerve damage and persistence of pain.

The literature on the potential role pre-injury exercise may play in reducing the consequences of peripheral neuropathy through neuroimmune responses has not yet been summarised. More insight is needed in what exercise type, program length, frequency, duration, intensity, and timing are necessary for improvements in pain-related and neuroimmune outcomes. For ethical reasons, invasive experimental research in this domain (e.g., immunohistochemistry of the nervous system) is not possible in humans. Research in animals may provide insight into processes that may be similar in humans. Therefore, the aim of this systematic review was to summarise the evidence on the effect of pre-injury exercise on neuroimmune, physiological and behavioural outcomes following experimentally-induced traumatic peripheral neuropathy in animals.

## 2 Methods

The review was reported according to the Preferred Reporting Items for Systematic Reviews and Meta-Analysis (PRISMA) guidelines (29) and registered at the International Prospective Register of Systematic Review (PROSPERO), CRD42021245899.

### 2.1 Literature search

MEDLINE (via PubMed), Web of Science and EMBASE databases were searched from inception until July 5, 2022. The search strategy was developed together with a research librarian (Alice Tillema) and consisted of three components (1) animals, (2) neuropathy and (3) exercise (**Appendix A**). Reference list of included studies and of relevant reviews were searched to identify other potentially eligible studies.

### 2.2 Study selection

Two independent researchers (MK, MS) performed the study selection by first screening title and abstracts and then full texts using the Rayyan screening tool (30). Disagreements were first discussed between the two independent researchers. A third reviewer (ILS or GSP) was consulted if a disagreement could not be resolved. Percentage agreement on study inclusion between the two reviewers was calculated. There were no restrictions on language nor publication date.

Studies that met the following criteria were considered for inclusion: (1) Design: (randomised) controlled trials of animal studies; (2) Population: animals with experimentally-induced traumatic peripheral neuropathy; (3) Intervention: active exercise program performed prior to nerve injury; (4) Control: no active exercise. Studies which investigated hereditary neuropathies or neuropathies acquired by disease or toxins (e.g., animal models for diabetes, rheumatoid arthritis, Guillain-Barré syndrome, systemic lupus erythematosus, or chemotherapy-induced peripheral neuropathy), or diseases or injuries to the central nervous system (e.g., Parkinson’s disease, spinal cord injury) were excluded. Studies were also excluded if the exercise intervention was passive (such as neural mobilisations or stretching (31)), consisted of electrical stimulation, or if the exercise was part of a multimodal intervention. Vehicle injections or sham graft injections near the injury site were considered co-interventions and these studies were therefore excluded. The outcome measures included were neuro-immune responses (i.e., neuroimmune responses are defined as processes or substances (such as neuropeptides, cytokines, gene expression and hormones) involved in interactions between the immune system and nervous system.), other physiological reactions (e.g., muscle or nerve morphometrics or behavioural outcomes (e.g., mechanical allodynia).

### 2.3 Data extraction

Data were collected by two reviewers independently (MK, PT). Details regarding bibliographic information (author and year), study design, animal model (species, strain, sex, age and weight), neuropathy model, exercise as an intervention (type, program length, frequency, duration, intensity, and mode) and all types outcomes were extracted from each paper. Outcomes were categorised into primary, i.e., measures related to neuroimmune responses, or secondary outcomes, i.e., other physiological or behavioural outcomes.

For all neuro-immune responses and other outcome measures, continuous data and/or percentages were extracted as mean, standard deviation, and number of animals. If the standardised error of the mean (SEM) was provided, then these values were recalculated to standard deviations. Authors of selected articles were contacted if data could not be extracted directly from the article or if the outcome data was incomplete (e.g., unclear number of animals used). If authors did not respond after two attempts, a digital screen ruler (Universal Digitizer 3.8, AVPSoft.com) was used by two independent review authors (MK, PT) to extract data from the graphs.

### 2.4 Assessment of risk of bias

The methodological quality of all included studies was evaluated by two independent reviewers (MK, PT) using the Systematic Review Center for Laboratory Animal Experimentation’s (SYRCLE) risk of bias tool for animal studies (32). The tool contains ten items related to selection bias, performance bias, detection bias, attrition bias, reporting bias and other bias. Each item is answered with ‘yes’ (i.e., low risk), ‘no’ (i.e., high risk), and ‘unclear’ (i.e., not enough information to determine the risk of bias). Discrepancies were first discussed between the two reviewers. If agreement could not be reached, a third reviewer (GSP) was consulted. Percentage agreement between the two reviewers was calculated.

### 2.5 Synthesis of results

The effects of pre-injury exercise on the primary outcome of neuroimmune responses and secondary outcomes of other physiological or behavioural outcomes were analysed using Comprehensive Meta-Analysis (CMA version 3). Standard mean differences (SMD) and 95% confidence intervals (95%CI) for each individual comparison were calculated with Hedges g correction (33). When group size was reported as a range (e.g., 8-10), the median value was used for meta-analysis. When a study contained multiple experimental groups, the control group size was corrected for the number of comparisons made (n/number of comparisons) (34, 35).

Meta-analyses in animal studies are more exploratory compared to those in human research, as direction of the effects is more meaningful than the size (35). In this review, meta-analyses were performed using a random effects model when a specific outcome was measured in at least two independent studies in the same anatomical location. Results from the meta-analyses were presented in forest plots and a pooled estimate was calculated. Neuroimmune outcomes measured in only one study, were also presented on a forest plot but without a pooled estimate. Vote counting was conducted for the neuroimmune outcomes by reporting the number of significant comparisons; results were cautiously interpreted. For repeated measurements at different time points within a study, the overall effect of pre-injury exercise was first presented using the largest recorded effect size, regardless of time, from each study. Statistical heterogeneity between studies was estimated by visual inspection and I^2^.

Pre-specified subgroup analyses included the comparison of species (e.g., rats versus mice), neuropathic pain model, exercise type (aerobic versus anaerobic). A *post-hoc* subgroup analysis was performed to explore the effect of timing of measurement post injury (week post injury). Subgroup analyses results were only interpreted if there were at least 10 comparisons per subgroup. To take into account the between study variation in the subgroup analyses, Comprehensive Meta-Analysis (CMA version 3) was used as it estimates the variation across all studies. To assess the publication bias, funnel plots and Trim and Fill analyses were planned for each outcome containing at least 10 studies (36). Lastly, to assess the robustness of the results, sensitivity analyses were planned to examine the effect of including only the low risk of bias compared to the complete analyses.

## 3 Results

### 3.1 Study selection

**Figure 1:**
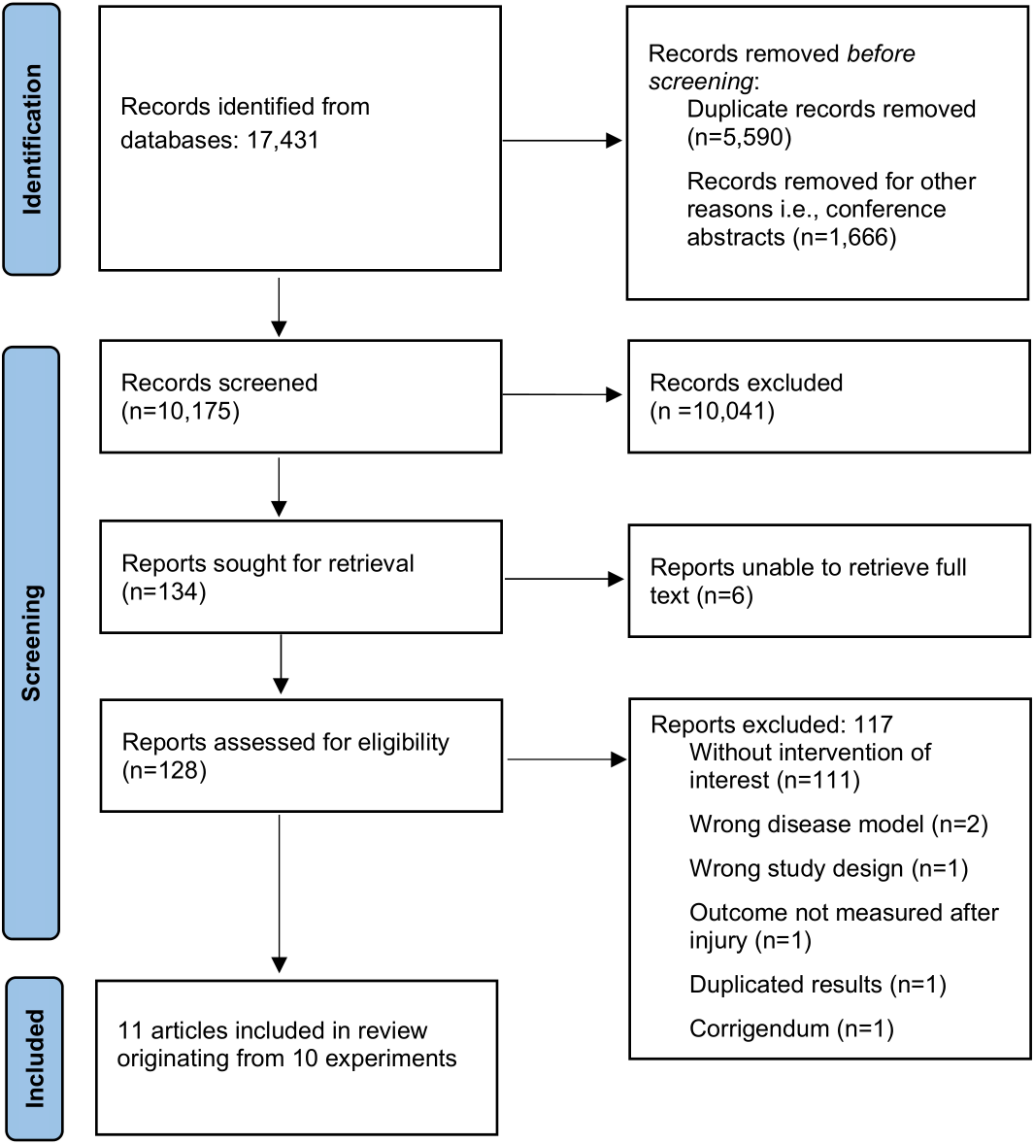
Flow chart of study selection procedure.

The flow of study selection is shown in **Figure 1**. The original search identified 17,431 records. After title and abstract screening, 128 full texts were assessed for eligibility, of which 11 articles originating from 10 experiments were included in the review (37–47). Reference lists of included studies and of relevant reviews (9, 48) did not reveal additional studies. Overall, agreement on study inclusion between the two reviewers before deliberation was 96.1%.

### 3.2 Study characteristics

The eleven articles, including a total of 103 independent comparisons and 167 animals, were published between 1998 and 2022 (37–47). All but one study (42) used adult, male rodents; with Wistar rats being the most common species used (6/11 studies) (37, 39, 44–47), followed by Sprague-Dawley rats (3/11 studies) (40, 42, 43) and mice (2/11 studies) (38, 41) All neuropathic pain models were performed by injuring the sciatic nerve, seven included studies used a chronic constriction injury (CCI) model (37, 39–43, 46), while four studies used a nerve crush model (38, 44, 45, 47). The exercise programs administered before the lesion mostly consisted of aerobic exercise (9/11 studies), such as running (38, 40–43, 46) or swimming (37, 39, 47), but also included aquatic resistance training programs (44, 45). The primary outcome, neuroimmune responses, such as cytokines, neurotrophic factors, microglia, and antioxidants, were measured in six studies (38, 40–43, 46). However, one study (41) did not analyse neuroimmune responses in the non-exercise control group, thus was excluded in the analyses for neuroimmune responses. In regards to the secondary outcomes, five studies (37, 38, 40, 44, 45) assessed other physiological outcomes, such as muscle or nerve morphometric. Behavioural outcomes, such as mechanical allodynia measured with von Frey filaments or sciatic function index (SFI), were measured in nine studies (38, 39, 41–47). A summary of the basic characteristics of the eleven included studies is presented in **Table 1**. All measured outcomes are listed in **Appendix B**.

**Table 1:** Characteristics of included studies.

Reference	Species	Sex	Age (weeks)	Weight (g)	Injury model	Exercise type	Exercise Program	Exercise intensity	Control intervention	Outcomes
**Artifon** **et al. 2013 (** 37)	Wistar rats n=6/group	Male	14 ± 2	413 ± 49	CCI	Swimming	10 min/day 5 days/week for 6 weeks	1. “Low” 2. Progressive:10min increase per week	Water exposure for 30 seconds	Soleus muscle fibre CSA, muscle fibre diameter, and muscle weight†
**Bertolini et al., 2011 (** 39)	Wistar rats n=6/group	Not reported	Not reported	413 ± 49	CCI	Swimming	10 min/day 3 days/week for 6 weeks	1. “Low” 2. Progressive:10min increase per week	Water exposure less than 1 minute	Functional disability test (time holding hind paw) †
**Bobinski et al.** **2011 (** 38)	Swiss Mice n=8/group	Male	8-9	25-35	Sciatic nerve crush	Treadmill running	30 min/day 5 days/week for 2 weeks	Low intensity: 10m/min	Motionless treadmill exposure	IL-1β, TNF-α, IL-6R, IL-10, total # of nerve fibres, nerve fibre density, nerve fibre diameter, axon diameter, myelin sheath thickness, G-ratio, SFI mechanical allodynia (von Frey), SSI†, grip force†, and cold hypersensitivity†
**Campos** **et al. 2018 (** 40)	Sprague-Dawley rats n=8-12/group	Male	Not reported	250-300	CCI	Treadmill running	60 min/day 5 days/week for 4 weeks	Moderate intensity: 60% maximum speed	Sedentary	αβ crystallin*, HSP27*, HSP90*, plantaris muscle fibre CSA*, LC3-I*†, LC3-II*†, p62*†, PolyUb*†, Carbonyl*†, caspase-3*†, BCL2*†, and muscle force ratio*†
**Cobianchi et al., 2010 (** 41)	CD1 Mice n=11/group	Male	Not reported	40-45	CCI	Treadmill running	TTE 5 days/week for 2 weeks	High intensity: 20 cm/s increased with 2 cm/s every 5 minutes until exhaustion	Sedentary	Mechanical allodynia (von Frey), CD11b†, GFAP†, and SSI†
**Grace** **et al.** **2016 (** 43)	Sprague-Dawley rats n=6/group	Male	10-12	Not reported	CCI	Wheel running	Voluntary for 6 weeks	Voluntary	Locked wheel	IL-10, IL-1β, Nitrite, NLRP3, p65, GLT-1, P2X4R, p38, BDNF, Iba-1, CCL2, CD11b, ATF3, Arg-1, iNOS, CCL3, CXCL1, and mechanical allodynia (von Frey)
**Green-Fulgham et al., 2022 (** 42)	Sprague-Dawley rats n=12-16/group	Male & Female	10	Not reported	CCI	Wheel running	1. Voluntary for 6 weeks 2. Voluntary for 3 weeks	Voluntary	Sedentary (single-housed, standard cage)	Nitrotyrosine, iNOS NOX2, superoxide dismutase 1, superoxide dismutase 2, heme oxygenase 1, heme oxygenase 2, Nrf2, and mechanical allodynia (von Frey)
**Kakihata et al.** **2016‡** (44)	Wistar rats n=5/group	Male	8	314 ± 23	Sciatic nerve crush	Resistance aquatic jumping exercise	3 days/week for 3 weeks	Low progressed to moderate intensity: - 2 sets of 10 jumps progressed to 4 sets - Load of 50% of animal’s weight	Sedentary	Nerve fibre diameter, axon diameter, myelin sheath thickness, G ratio, nerve fibre density, total # of nerve fibres, SFI, blood vessel density†, nerve connective tissue† and cell nuclei density†
**Malanotte et al.** **2017‡** (45)	Wistar rats n=5/group	Male	8	314 ± 23	Sciatic nerve crush	Resistance aquatic jumping exercise	3 days/week for 3 weeks	Low progressed to moderate intensity: - 2 sets of 10 jumps progressed to 4 sets - Load of 50% of animal’s weight	Sedentary	Soleus muscle fibre CSA, muscle fibre diameter, mechanical allodynia (von Frey) and muscle connective tissue†
**Safakhah et al., 2017 (** 46)	Wistar rats n=6-9/group	Male	Adult	200 ± 20	CCI	Treadmill running	30 min 5 days/week for 3 weeks	High intensity: 16m/min	Sedentary	TNF-α*, MDA*, FRAP*, mechanical allodynia* (von Frey), and heat hypersensitivity†
**van Meeteren et al.** **1998 (** 47)	Wistar rats n=10/group	Male	Not reported	140-160	Sciatic nerve crush	Swimming	3x3-min/day 7 days for 1 week	High intensity: 20m/min	Swam 6 meters (~18 seconds) for 3 days	SFI and foot reflex withdrawal test†

*Data extracted using a digital ruler. †Outcome measured only in one study thus, not analysed in the meta-analyses. ‡Studies used same experimental sample of animals but reported different outcomes. SFI, sciatic function index; SSI, sciatic static index; CCI, chronic constriction injury; CSA, cross-sectional area; MDA, malondialdehyde; FRAP, ferric reducing ability of plasma.

### 3.3 Risk of bias within studies

**Figure 2:**
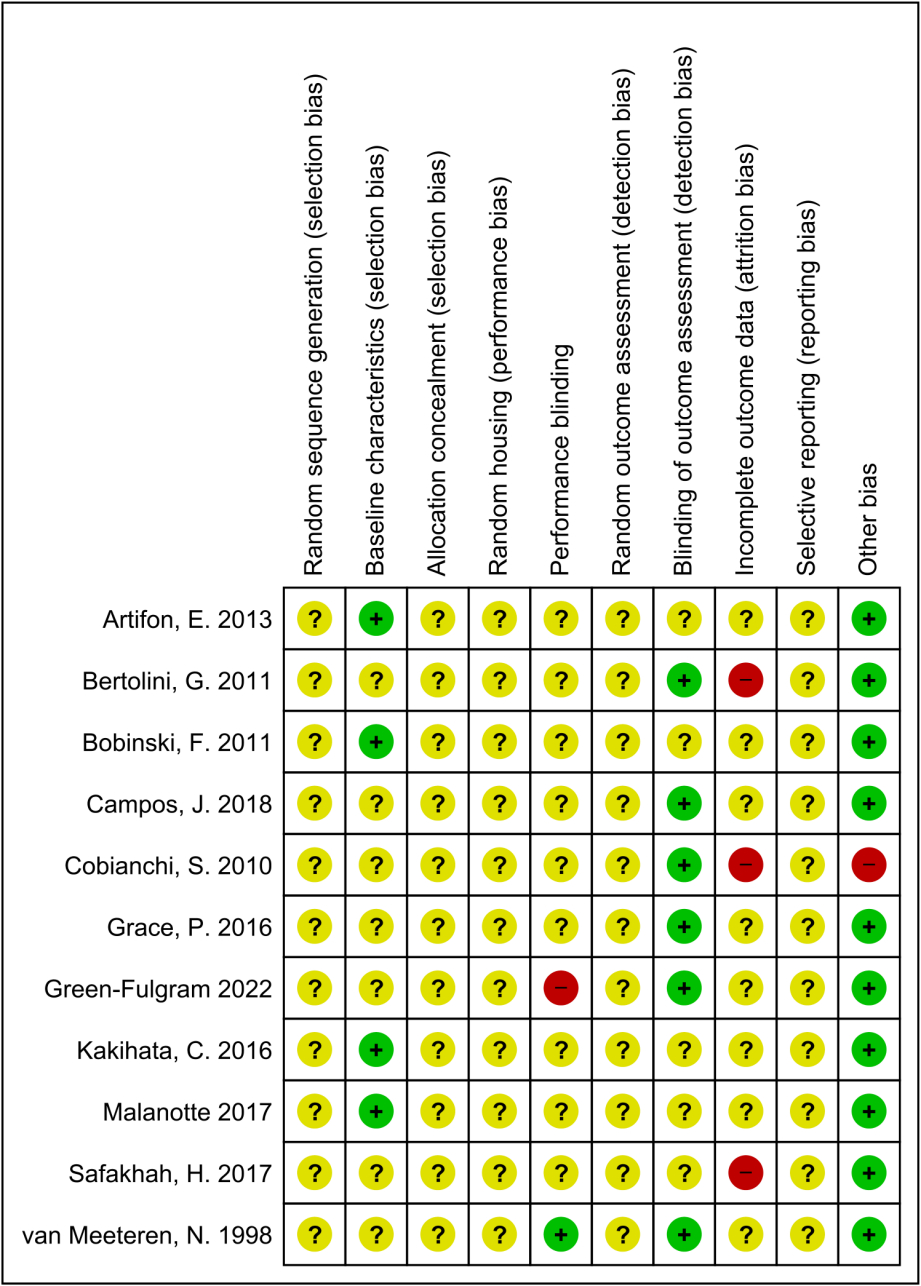
Risk of bias summary. Review authors’ judgement for each risk of bias item. The green ‘+’ means low risk, red ‘-’ means high risk, and yellow ‘?’ means unclear.

**Figure 2** summarises the results of the risk of bias assessment for each study. Regarding five of the risk of bias items: random sequence generation, allocation sequence, random housing, random outcome assessment, and selective outcome reporting, all included studies showed an unclear risk of bias, because essential details regarding the methodology was not available. For the rest of the risk of bias items, the percentage marked as ‘unclear risk’ are as follows: baseline characteristics (64%), performance blinding (82%), blinding of outcome assessment (45%), and incomplete outcome data (73%). Low risk of bias was scored for all but one included study for the question, “Was the study apparently free of other problems that could result in high risk of bias? The high risk scored for this item pertained to excluding mice that refused to run. The percentage of agreement between the reviewers was 82%; differences between raters were mainly due to interpretation differences.

### 3.4 Effect of pre-injury exercise on neuroimmune responses following experimentally-induced traumatic peripheral neuropathy

**Figure 3:**
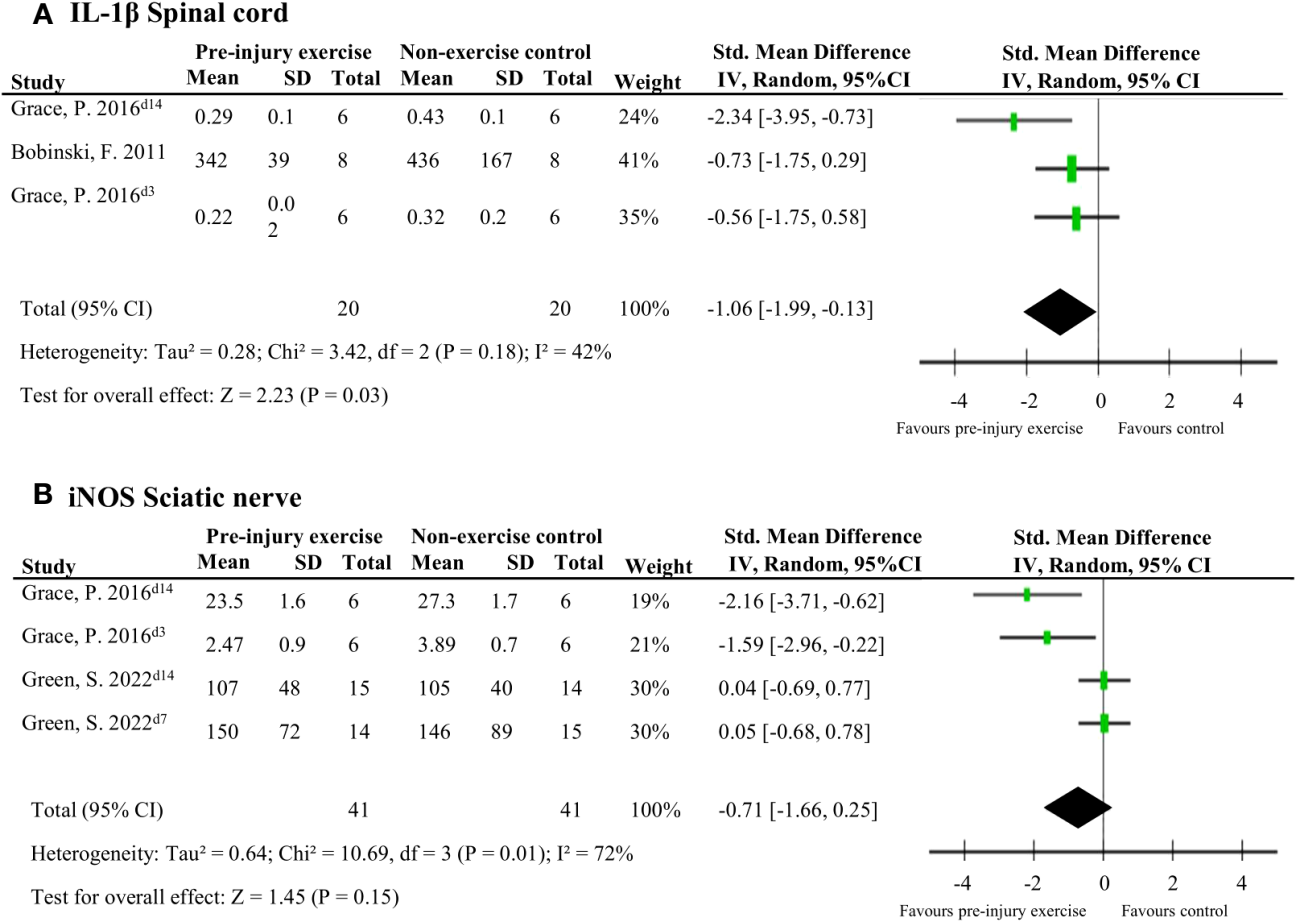
The general effect of pre-injury exercise on various pro-inflammatory and anti-inflammatory neuroimmune outcomes in animal models of experimentally-induced traumatic peripheral neuropathy. **(A)** Meta-analysis of the effect of pre-injury exercise on IL-1β. **(B)** Meta-analysis of the effect of prior exercise on iNOS. For neuroimmune outcomes measured in the same study and location, but different timepoints, the day post injury is listed behind study identification in superscript.

Five studies (92 rodents) evaluated 72 different neuroimmune responses measured at six different locations (spinal cord, cerebrospinal fluid, dorsal root ganglion, sciatic nerve, muscle and blood/serum) (38, 40, 42, 43, 46). Neuroimmune responses were measured on various days post-injury: 3,7,14, 15 or 26. When a neuroimmune response was measured in the same location in multiple comparisons, a meta-analysis was conducted. Meta-analysis on levels of IL-1β in the spinal cord ranging from 3-15 days post-injury showed that pre-injury exercise compared to non-exercise control significantly reduced levels of IL-1β (pooled data, 2 studies, 3 comparisons, n=40 rodents, SMD: -1.06, 95%CI: -1.99 to -0.13) (38, 43) (**Figure 3A**). Two other studies (42, 43) showed that pre-injury exercise did not significantly reduce levels of iNOS at the sciatic nerve compared to non-exercise control (pooled data, 2 studies, 4 comparisons, n=82 rodents, SMD: -0.71, 95%CI: -1.66 to 0.25) (**Figure 3B**). iNOS is an enzyme that produces nitric oxide under stress conditions and is a common marker for M1 macrophage activation (49–51). Due to the low power of these meta-analyses, no strong conclusions can be drawn.

**Figure 4:**
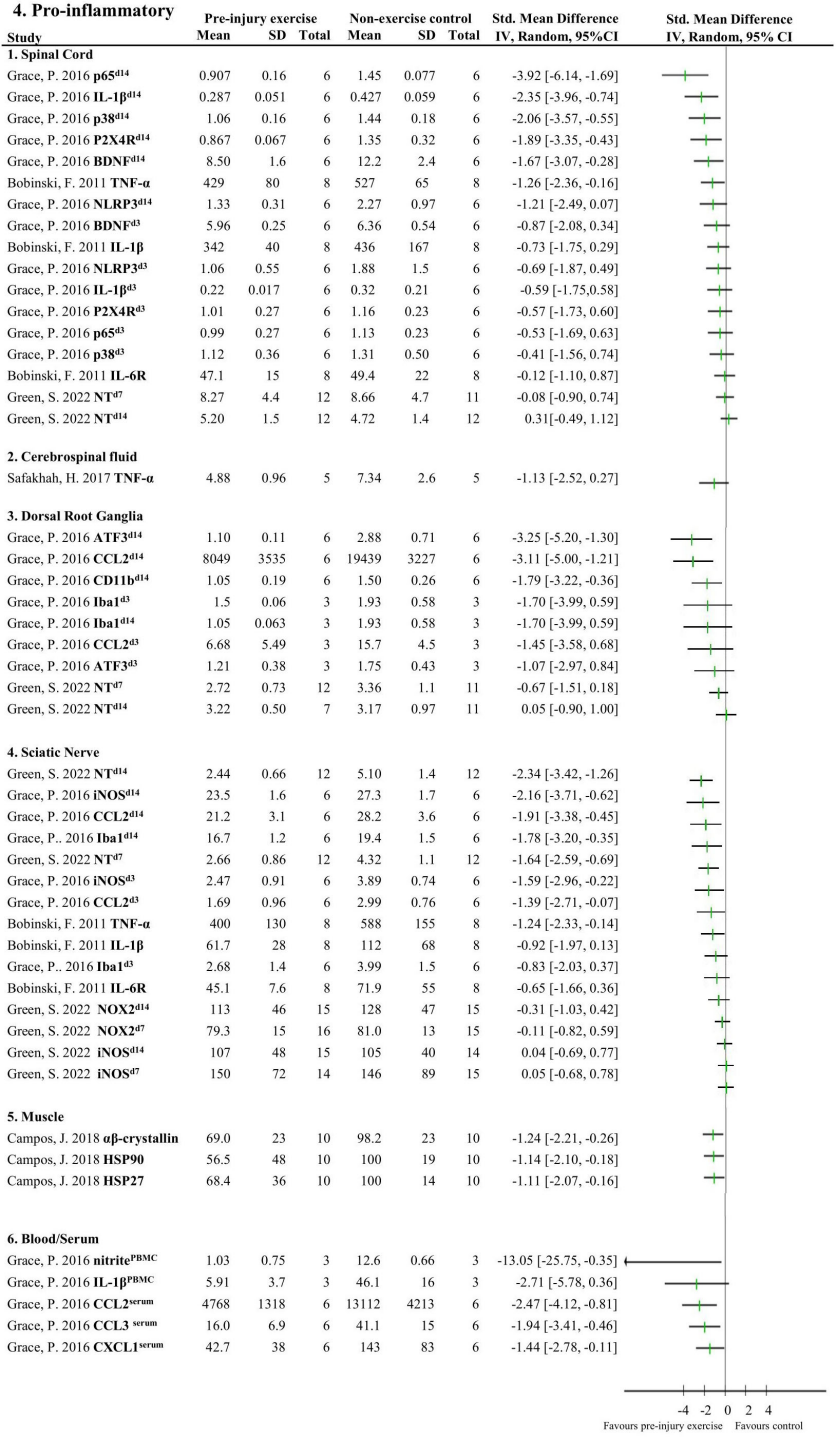
Overview of all reported pro-inflammatory neuroimmune responses. Favours pre-injury exercise implies a reduction in pro-inflammatory neuroimmune markers. Outcomes are categorised by anatomical location. For neuroimmune outcomes measured in the same study and location, but different timepoints, the day post injury is listed behind study identification in superscript. p65, nuclear factor NF-kappa-B p65 subunit; IL-1β, interleukin 1 beta; p38 mitogen-activated protein kinase; P2X4R, P2X4 receptor; BDNF, brain-derived neurotrophic factor; TNF-α, tumor necrosis factor alpha; NLRP3 inflammasome; IL-6R, interleukin-6 receptor; NT, nitro tyrosine; ATF3, activating transcription factor 3; CCL2, chemokine (C-C motif) ligand 2, CD11b (macrophage marker); Iba1, ionized calcium binding adaptor molecule 1 (microglia activation marker); iNOS, inducible nitric oxide synthase; NOX2, NAPDH oxidase 2; HSP, heat shock protein; PBMC, peripheral blood mononuclear cells; CXCL1, chemokine (C-X-C motif) ligand 1.

**Figure 5:**
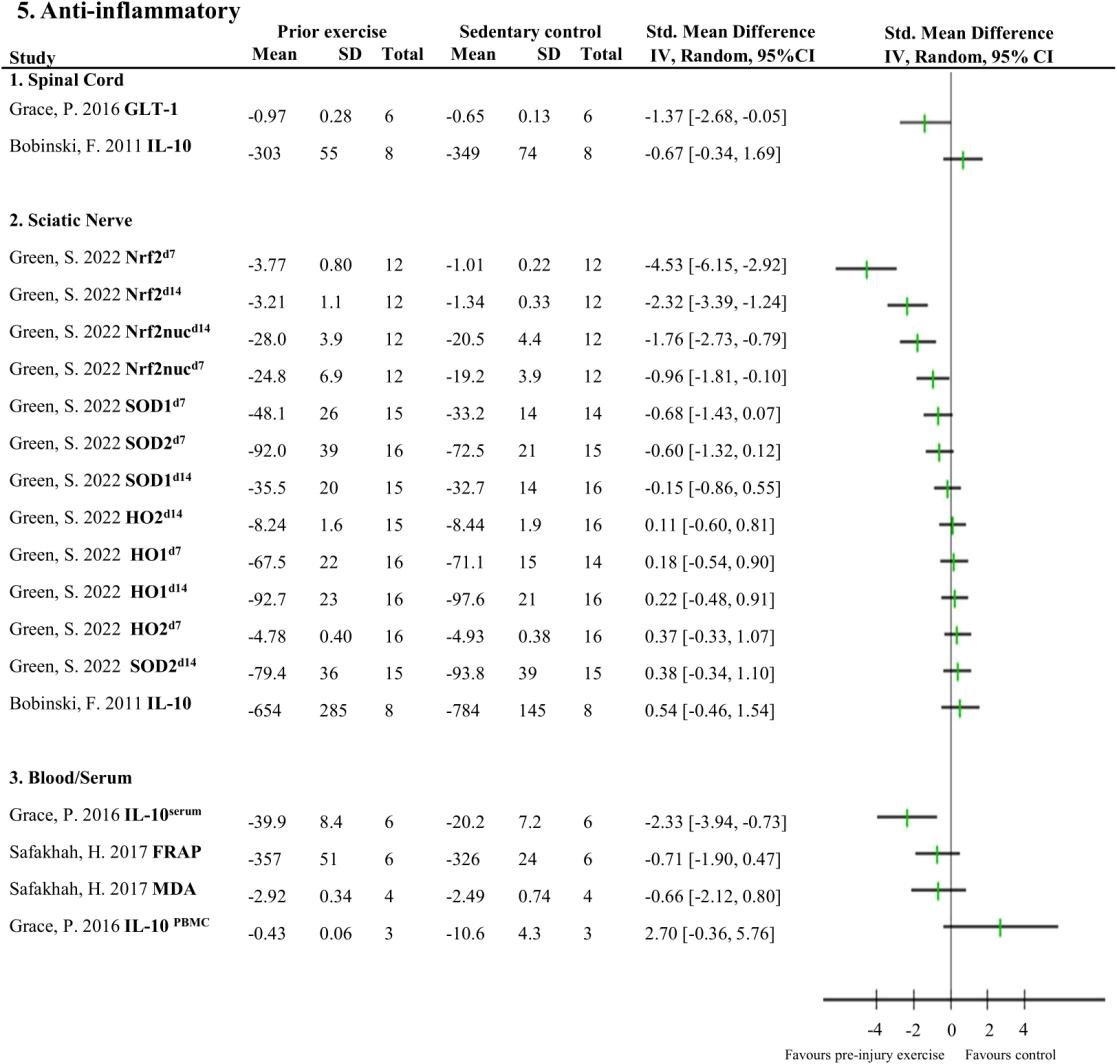
Overview of all reported anti-inflammatory neuroimmune responses Favours pre-injury exercise implies an increase in anti-inflammatory neuroimmune markers. Outcomes are categorised by anatomical location. For neuroimmune outcomes measured in the same study and location, but different timepoints, the day post injury is listed behind study identification in superscript. GLT-1, glutamate transporter 1; IL-10, interleukin 10; Nrf2, nuclear factor E2-related factor 2 nuclear translocation; Nrf2nuc, nuclear factor E2-related factor 2 nuclear fractions; Arg-1, arginase 1; SOD, superoxide dismutase; HO, heme oxygenase; FRAP, ferric reducing antioxidant power; MDA, malondialdehyde.

**Figures 4**, **5** provide an overview of all reported neuroimmune responses, for pro-inflammatory (**Figure 4**) and anti-inflammatory (**Figure 5**) responses. This forest plot shows the direction of the effect of pre-injury exercise on neuroimmune responses per anatomical location following experimentally-induced traumatic peripheral neuropathy. Vote counting revealed that for the pro-inflammatory neuroimmune responses (**Figure 4**), pre-injury exercise significantly improved 24 of the 50 comparisons across the six different locations. Of the 22 comparisons evaluating anti-inflammatory neuroimmune responses, pre-injury exercise significantly improved 6 responses across the different locations (**Figure 5**).

### 3.5 Effect of pre-injury exercise on nerve morphometrics following experimentally-induced traumatic peripheral neuropathy

**Figure 6:**
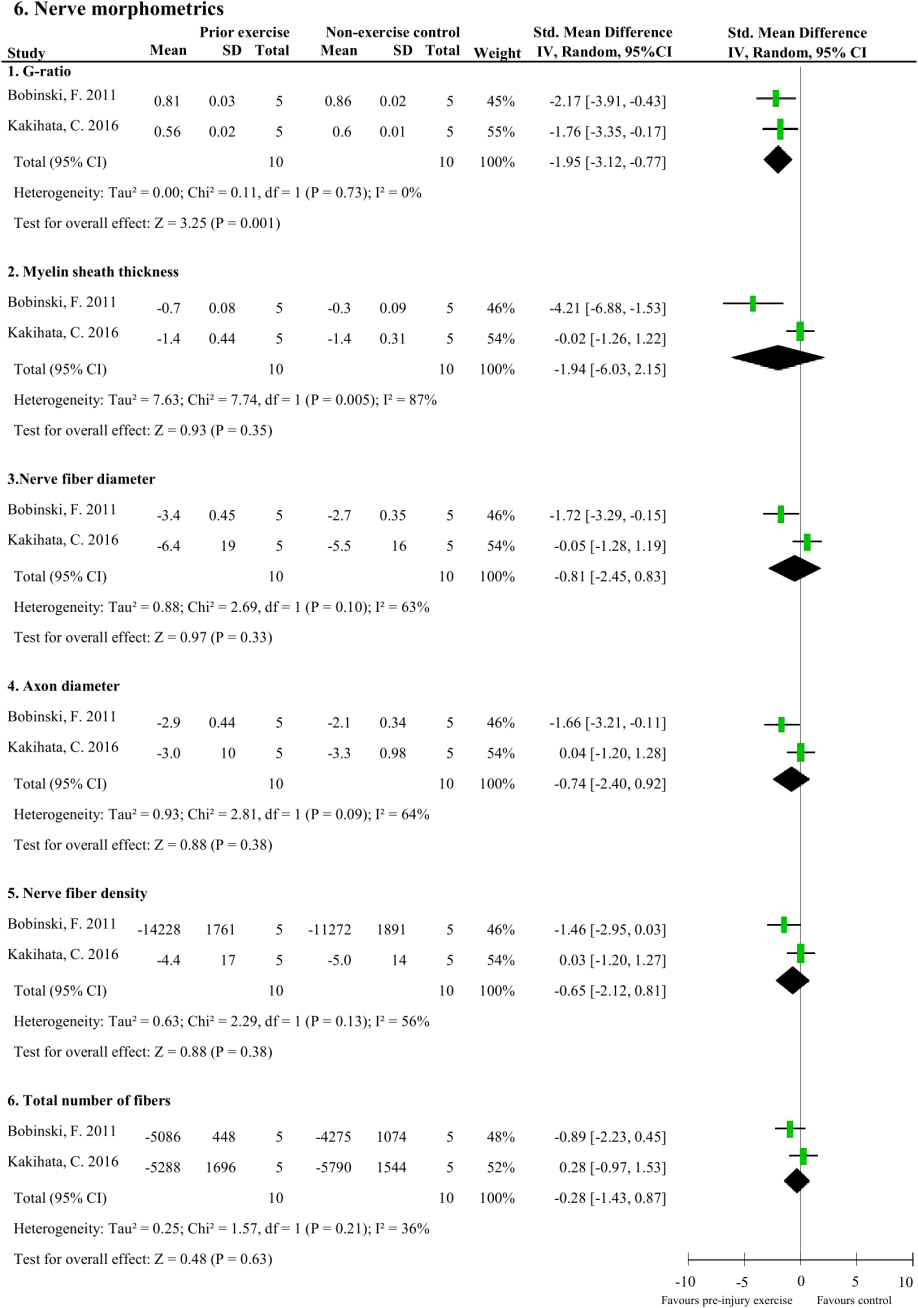
The effect of pre-injury exercise on six different nerve morphometric outcomes following experimentally-induced traumatic peripheral neuropathy. Favours pre-injury exercise implies an increase in outcome values, except for G-ratio where a reduction in outcome is valued.

Meta-analyses could be performed on two studies (20 rodents) which measured six nerve-related morphometrics (38, 44). Exercise before experimentally-induced traumatic peripheral neuropathy had a favourable effect on the sciatic nerve’s G-ratio (i.e., a measure of the functional and structural index of optimal axonal myelination (52), determined by the inner axon radius relative to the outer, myelinated, axon radius) compared to non-exercise control (pooled data, 2 studies, n=20 rodents, SMD: 1.95, 95%CI: 0.77 to 3.12) (**Figure 6.1**) (38, 44). Pre-injury exercise was not different than non-exercise control on other nerve-related morphometrics, such as myelin sheath thickness (pooled data, 2 studies, n=20, SMD: 1.94, 95%CI: –2.15 to 6.03), nerve fibre diameter (pooled data, 2 studies, n=20, SMD: 0.81, 95%CI: -0.83 to 2.45), axon diameter (pooled data, 2 studies, n=20, SMD: 0.74, 95%CI: -0.92 to 2.40), nerve fibre density (pooled data, 2 studies, n=20, SMD: 0.65, 95%CI: -0.81 to 2.12), or total number of nerve fibres (pooled data, 2 studies, n=20, SMD: 0.28, 95%CI: -0.87 to 1.43) (**Figure 6.2-6**) (38, 44).

### 3.6 Effect of pre-injury exercise on muscle morphometrics following experimentally-induced traumatic peripheral neuropathy

There were two muscle morphometric measures that were assessed by two studies (48 rodents). Muscle fibre cross-sectional area of the soleus (37, 45) and plantaris (40) muscle following peripheral neuropathy induction was significantly larger with pre-injury exercise (pooled data, 3 studies, 4 comparisons, n=48 rodents, SMD: 0.91, 95%CI: 0.27 to 1.54) compared to non-exercise control (**Figure 7.1**). Pre-injury exercise was not different than the non-exercise control in muscle fibre diameter (pooled data, 2 studies, n=28, SMD: 0.76, 95%CI: -0.07 to 1.60) (**Figure 7.2**) (37, 45).

**Figure 7:**
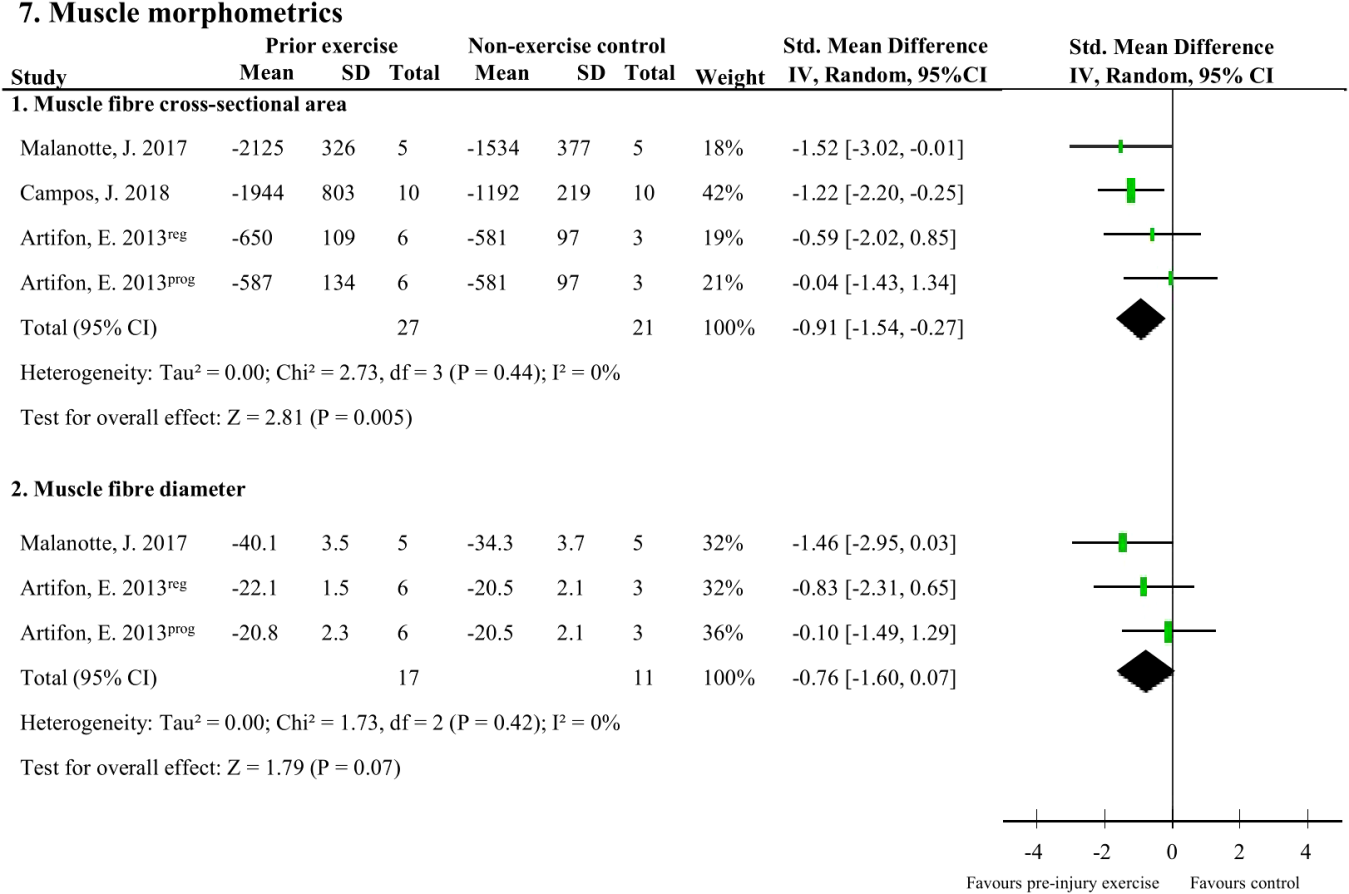
The effect of pre-injury exercise on two outcomes related to muscle morphometrics following experimentally-induced traumatic peripheral neuropathy. Favours pre-injury exercise implies an increase in outcome values. Artifon et al., 2013 had two experimental groups: the progressive swimming program is labelled “prog” and the regular aerobic swimming is labelled “reg”.

### 3.7 Effect of pre-injury exercise on behavioural/functional outcomes at the hind paw following experimentally-induced traumatic peripheral neuropathy

**Figure 8:**
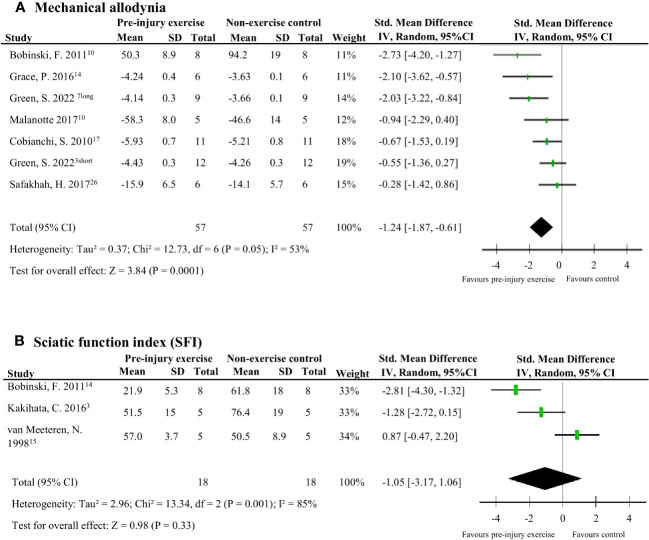
The effect of pre-injury exercise on behavioural outcomes following experimentally-induced traumatic peripheral neuropathy. As both outcomes, mechanical allodynia and sciatic function index (SFI) were repeated measurements taken at multiple time points, only the largest effect per study are presented. Days post injury listed behind study identification in superscript. **(A)** The effect of prior exercise on mechanical allodynia. Favours pre-injury exercise implies an improvement in mechanical allodynia. Green et al., 2022 had two experimental groups: a short duration program of 3 weeks labelled “short” and a long duration program of 6 weeks labelled “long”. **(B)** The effect prior exercise on SFI. Favours pre-injury exercise implies an improvement in SFI scores.

Nine studies (124 rodents) evaluated behavioural outcomes after peripheral neuropathy induction (38, 39, 41–47). Meta-analyses could be performed on two outcomes: mechanical allodynia and SFI measured at the hind paw. Mechanical allodynia scores, measured with von Frey filaments, were significantly better in the pre-injury exercise group compared to and non-exercise control (pooled data, 6 studies, 7 interventions, n=114, SMD -1.24, 95%CI: -1.87 to -0.61) (**Figure 8A**) (38, 41–43, 45, 46)[38]. There was no difference however, between pre-injury exercise compared to non-exercise control in SFI scores (pooled data, 3 studies, n=36, SMD: 1.05, 95%CI: -1.06 to 3.17) (**Figure 8B**) (38, 44, 47).

### 3.8 Subgroup analysis

**Figure 9:**
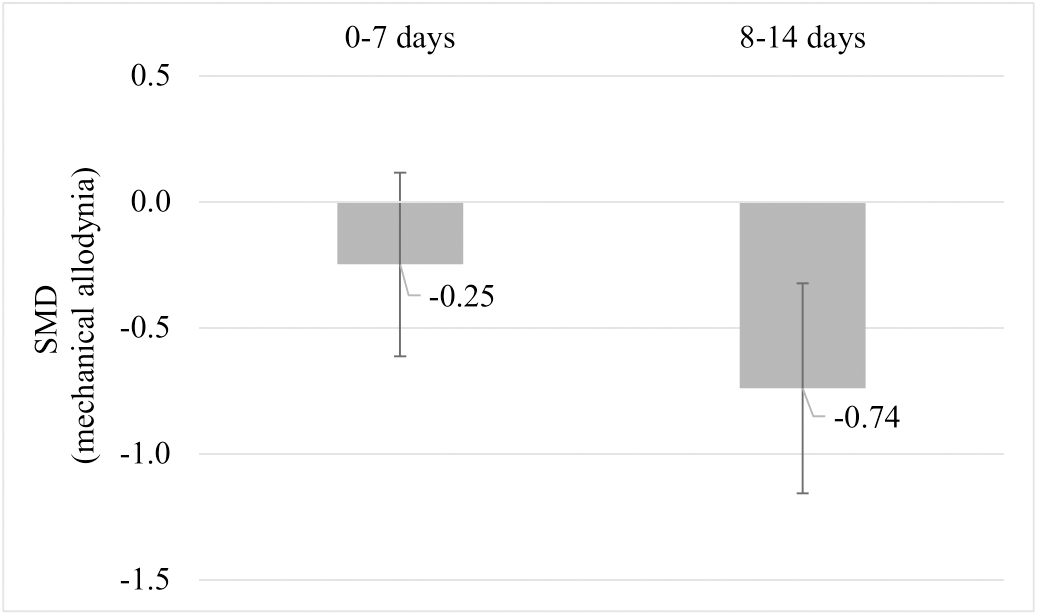
*Post hoc* subgroup analysis: The effect of pre-injury exercise on mechanical allodynia grouped by week post injury following experimentally-induced traumatic peripheral neuropathy. Negative values favour pre-injury exercise. The error bars represent the 95% confidence intervals. The results from subgroup analysis were only presented when subgroups contained data from at least 10 independent comparisons.

Due to the limited number of studies, none of the planned subgroup analysis could be performed. A *post-hoc* subgroup analysis for mechanical allodynia was performed comparing mechanical allodynia measurements recorded per week post-injury. Comparing the subgroups of mechanical allodynia per week post-injury, pre-injury exercise reduced mechanical allodynia at week 2 but not significantly at week 1 post injury (**Figure 9**). Sub-groups of mechanical allodynia measured during week 3 and 4 had less than 10 comparisons but results can be found in **Appendix C**. These results suggests that the timing of outcome measurement may influence the effect of pre-injury exercise on mechanical allodynia. However, due to the low number of studies in each subgroup we have to interpret these findings carefully.

### 3.9 Publication bias and sensitivity analysis

Due to the low number of studies measuring the same outcome, publication bias could not be investigated. Furthermore, proposed sensitivity analyses could not be performed as the risk of bias was unclear for the majority of the included studies in all risk of bias domains.

## 4 Discussion

This systematic review summarises the results of 11 studies that examined the influence of pre-injury exercise on neuroimmune outcomes, physiological responses, and behavioural outcomes following experimentally-induced peripheral neuropathy in animal models. Most studies evaluated aerobic exercise interventions and used a sciatic nerve, chronic constriction injury (CCI) model. Seventy-two different neuroimmune outcomes (mostly pro-inflammatory) were measured in five included papers in different anatomical locations. Meta-analyses could only be conducted for IL-1β (spinal cord) and iNOS (sciatic nerve) and showed exercise prior to experimentally-induced traumatic neuropathy significantly reduced IL-1β levels but not iNOS. All the other neuroimmune outcomes were only measured once. Vote counting revealed a reduction in 23 pro-inflammatory neuroimmune outcomes (such as cytokines, chemokines, and macrophage/microglial markers) and an increase in 6 anti-inflammatory neuroimmune outcomes (such as transcriptional antioxidant response regulator, Nrf2, and glutamate transporter-1). Physiological responses, such as the G-ratio of the nerve and the muscle fibre cross-sectional area of the distally innervated muscle, were improved in the pre-injury exercise group compared to non-exercise control. Behavioural outcomes, such as mechanical allodynia improved in the pre-injury exercise group compared to non-exercise control. The *post-hoc* subgroup analysis for mechanical allodynia suggests that the timing of outcome measurement may influence the effect of pre-injury exercise on mechanical allodynia, but results from small subgroups. The results of this review suggest a potential neuroprotective and immunoregulatory effect of an exercise regime before sustaining a peripheral neuropathy.

### 4.1 Effects of pre-injury exercise

Experimentally-induced traumatic peripheral neuropathy provokes an immune response that requires an intricate balance of pro-inflammatory and anti-inflammatory mechanisms (14, 53). Current evidence suggests that persistent neuropathic pain is the result of the balance being tipped in favour of pro-inflammatory mechanisms (14, 16). Regular exercise is known to induce a transient anti-inflammatory environment, locally within the tissues and systemically (22, 23, 54). In this review, most included studies focused on the reduction of pro-inflammatory responses, such as IL-1β or BDNF related transcription factors (e.g., p65, p38, and P2X4R) other cytokines and chemokines, macrophage/microglia markers (CD11b and Iba1), and neuronal damage factor ATF3. Meta-analyses could only be performed on 2 neuroimmune markers (spinal cord IL-1β and sciatic nerve iNOS). Meta-analysis on levels of IL-1β from two studies (n=40 rodents) showed that pre-injury aerobic exercise significantly reduced levels of IL-1β compared to non-exercise control. A potential explanation for the lack of significant results in the meta-analysis for iNOS could be a result of too few studies (n=2, n=41 rodents) leading to type II error. Pre-injury exercise promoted anti-inflammatory responses including elevated levels of GLT-1 and transcriptional antioxidant response regulator, Nrf2. Activation of Nfr2 seems to be a promising mechanism as it attenuated CCI-induced neuropathic pain via induction of PGC-1α-mediated mitochondrial biogenesis in the spinal cord (55). Additionally, Nrf2 activation reduces oxidative stress and neuroinflammation leading to a reduction in pain and delays the onset of pain in various animal models (56). Thus, pre-injury exercise may influence different neuroimmune signals on both sides of the inflammatory balance to prevent the sustained hyperinflammation as seen in non-exercise animals.

This review also demonstrates that exercise exerts its effects not only locally near the injury site (sciatic nerve), but also in the dorsal root ganglia and spinal cord. Unfortunately, the effect of pre-injury exercise on the brain was not evaluated in the included studies, despite evidence supporting benefits of exercise on the brain, astrocyte function, and specific regions important in pain modulation (24, 57, 58). Then again, the activation of microglia in the dorsal horn of the spinal cord is a critical contributor to the initiation and maintenance of neuropathic pain (11, 17, 59–62). Therefore, it is encouraging that in this review, pre-injury exercise influences several mechanisms in the peripheral and central nervous system involved in the development and repercussions of neuropathic pain.

Unexpectedly, no overall effect was seen for the functional behavioural outcome, SFI. While for the behavioural outcome of mechanical allodynia, the pre-injury exercise group had significantly less sensitivity to touch compared to the non-exercise control. Typically, mechanical allodynia in rodents occurs 3-7 days after CCI and lasts to about 35 days (63–65). In a *post hoc* subgroup analysis, mechanical allodynia results were grouped by week post injury. The results provide some insight on the sequela of peripheral nerve injury and shows that there might be a time effect but coming from very small groups. By grouping the repeated measures at different time points by time after injury in the *post-hoc* analyses, the pre-injury exercise group had significantly less mechanical allodynia at week 2, but not week 1. Interestingly, this is the same time period when most neuroimmune outcomes reported in this review were measured. This shared timing is most likely not a coincidence, as the severity of mechanical allodynia was recently correlated with the level of dorsal root ganglion inflammation (65). More research needs to be undertaken to understand the lasting effects of pre-injury exercise, whether its effect also lies in this time window of 1-2 weeks post neuropathic pain induction.

### 4.2 Limitations and steps forward

When interpreting the findings of this systematic review and meta-analyses, several limitations should be considered. The first and foremost is that the number of studies that could be analysed per outcome was very low. Thus, increasing the likelihood of imprecise effect estimates which influences the robustness and reliability of the conclusions that can be drawn from this systematic review. Much more research on the neuroimmune responses and neuroprotective effects of pre-injury exercise before the occurrence of traumatic peripheral neuropathy is urgently needed. In addition, the heterogeneity between the studies was large. Studies differed for example, in species used, exercise program duration, and exercise intensity. To account for anticipated heterogeneity, we used a random effects model and explored the suggested causes for between study heterogeneity by means of subgroup analysis.

Thirdly, risk of bias analysis revealed poor reporting of essential details related to the design and conduct of the included experiments. Consequently, in a majority of the studies, risk of bias could not be estimated. The lack of reporting important methodological details raises concerns about bias in the data and skewed results thus, hampering the ability to draw reliable conclusions from the included animal studies. Future preclinical studies should follow ARRIVE guidelines to ensure better transparency in their methodology and reporting (66, 67). The Enhancing Quality in Preclinical Data (EQIPD) framework may also be a tool to help pre-clinical researchers to decide which guidelines to follow (67).

Fourthly, in preclinical pain research the indirectness of the results should be considered (68) – in particular the indirectness of the animal models of neuropathic pain and the outcome measures of pain. The traumatic peripheral neuropathy models (sciatic CCI and nerve crush) used by included studies in this review have the ability to represent some certain neuropathic conditions in humans (e.g., sciatic, radiculopathy, chronic low back pain, and complex regional pain syndrome type II) (69). In terms of the development of pain-related hypersensitivity and underlying pathogenesis, these two models share many features (70). However, some dissimilarities may exist (e.g., different inflammatory reaction severity (70)). Outcomes measured in these neuropathic models such as, biological markers and behavioural tests, are indirect measures of human pain (71). Therefore, translation of the results to the clinical setting is not appropriate. Additionally, the results of this review cannot be extrapolated to female animals as all included studies used male rodents, except one (42). Sex differences are becoming more apparent in neuroimmune responses (72), biological mechanisms of pain maintenance after nerve injury (73), and even in the effectiveness of exercise dependent on the type (74–76). Thus, despite the fact that sex differences in pain sensitivity have been well established for decades (77, 78), the translatability of the results within the preclinical field are limited.

The aforementioned limitations make it difficult to draw conclusions on the mechanisms underlying the effect of pre-injury exercise on recovery following experimentally-induced traumatic peripheral neuropathy. However, this review does provide an overview of the neuroimmune outcomes/mechanisms that have been measured to date. Future research may use this as a guide in selecting neuroimmune targets. Researchers should also keep in mind other factors that could influence neuroimmune responses, for instance forced treadmill and swimming may increase stress biomarkers compared to voluntary exercise (24, 79). The subgroup analyses provide some insight for preclinical researchers analysing mechanical allodynia to be mindful of (or further investigate) the type of animal model used, the intensity of the exercise, the exercise program duration, and the timing of outcome assessment during the recovery process. Similar factors were also suggested in a brief narrative review on the overall effects of exercise on chronic pain (80). More specifically, voluntary exercise paradigms, exercise program duration, and the chronic pain animal model possibly contributed to the effectiveness of pre-injury exercise in rodent models of chronic pain (80).

This is the first review to focus on the effect of pre-injury exercise on recovery in terms of neuroimmune outcomes, physiological response, and behavioural outcomes following experimentally-induced traumatic peripheral neuropathy in animals. The findings in this systematic review suggest a potential neuroprotective and immunoregulatory effect of pre-injury exercise for the recovery after peripheral induced neuropathic pain. However, as most findings were based on single studies, more research is needed to increase the certainty of evidence. Future research could focus on a pro-inflammatory state resulting in a prolonged, exaggerated pain state and susceptibility to chronic pain being the consequence of non-exercise behaviour. On top of the many benefits, regular exercise seems to promote a normal healing process following experimentally-induced traumatic peripheral neuropathy.

## Author contributions

All authors contributed substantially to this systematic review, and are listed alphabetically: Conceptualisation, GS-P, MC, MK, and MS-K; methodology, CH, GS-P, IL, MC, MK, and MS-K; data curation CH, GS-P, MK, MS-K, and PT; writing—original draft preparation, MK; writing—review and editing, CH, GS-P, IL, MC, MK, MS-K, and PT; visualisation, CH, GS-P, MC, and MK; supervision, GS-P and MC; project administration, GS-P and MC; funding acquisition, GS-P. All authors contributed to the article and approved the submitted version.
